# β-Amyloid precursor protein (APP) and the human diseases

**DOI:** 10.3934/Neuroscience.2019.4.273

**Published:** 2019-10-29

**Authors:** Khue Vu Nguyen

**Affiliations:** 1 Department of Medicine, Biochemical Genetics and Metabolism, The Mitochondrial and Metabolic Disease Center, School of Medicine, University of California, San Diego, Building CTF, Room C-103, 214 Dickinson Street, San Diego, CA 92103-8467, USA; 2 Department of Pediatrics, University of California, San Diego, School of Medicine, San Diego, La Jolla, CA 92093-0830, USA

**Keywords:** human β-amyloid precursor protein, epigenetics, epistasis, alternative splicing, neurodevelopmental and neurodegenerative disorders, rare diseases and common and complex disorders, antisense drugs

## Abstract

Several pathophysiological functions of the human β-amyloid precursor protein (APP) have been recently proposed in different human diseases such as neurodevelopmental and neurodegenerative disorders including rare diseases such as autism, fragile X syndrome, amyotrophic lateral sclerosis, multiple sclerosis, Lesch-Nyhan disease; common and complex disorders such as Alzheimer's disease; metabolic disorders such as diabetes; and also cancer. APP as well as all of its proteolytic fragments including the amyloid-β (Aβ) peptide, are part of normal physiology. The targeting of the components of APP proteolytic processing as a pharmacologic strategy will not be without consequences. Recent research results highlight the impact of alternative splicing (AS) process on human disease, and may provide new directions for the research on the impact of the human APP on human diseases. The identification of molecules capable of correcting and/or inhibiting pathological splicing events is therefore an important issue for future therapeutic approaches. To this end, the defective APP-mRNA isoform responsible for the disease in cells and tissues appears as an ideal target for epigenetic therapeutic intervention and antisense drugs are potential treatment.

## 1. Introduction

The human β-amyloid precursor protein (APP): its structure, and its cellular roles as well as its proteolytic processing are in the focus of intensive research due to the central role of APP during the development of Alzheimer's disease (AD). AD is a degenerative brain disorder and is, in fact, the most common form of dementia. AD is characterized by a decline in faculties such as memory, language, problem-solving and other cognitive skills that affect a person's ability to perform everyday tasks. AD is characterized by two major pathological hallmarks: extracellular deposition of amyloid plaques of amyloid-β (Aβ) peptide originated from proteolysis of APP between neurons in the brain, and intracellular aggregates of neurofibrillary tangles of hyperphosphorylated tau proteins inside the neurons. AD is the main cause of dementia in more than 80% of geriatric population and it is expected that the present number of 46.8 million persons in the world suffering from dementia will reach a height of 74.7 million in 2030 and 131.5 million in 2050. For now, clinically validated treatments for AD remain confined to symptomatic interventions. Owing to the dramatic increase in the population as the year and age progresses, AD is often hired as life threatening as well as economic and social burden to the health-care system [1]. Recently, several pathophysiological functions of APP have been proposed in different human diseases such as neurodevelopmental and neurodegenerative disorders including rare diseases such as autism, fragile X syndrome (FXS), amyotrophic lateral sclerosis (ALS), multiple sclerosis (MS), Lesch-Nyhan disease (LND); common and complex disorders such as AD; metabolic disorders such as diabetes; and also cancer. The present mini-review provides an overview about the impact of APP on human diseases, and concludes with an opinion by emphasizing on the RNA-based therapy via antisense oligonucleotides to correct splicing defects.

## 2. Human APP and its impact on human diseases overview

### 2.1. APP structure and processing

APP is ubiquitously expressed in a broad spectrum of cell types including non-neuronal cells, and it is suggested to be involved in the growth of these cells [2]–[4], while the nature of APP has been mainly studied in neuronal cells due to its pathological significance in AD. The APP family in mammals consists of three members: APP, the APP-like protein-1 (APLP1) and the APP-like protein-2 (APLP2). APP gene is located on chromosome 21 (21q21.2-3), whereas APLP1 gene is located on chromosome 19 (19q13.12) and APLP2 gene is located on chromosome 11 (11q24.3). All three proteins are type I transmembrane proteins with similar structure and membrane topology, and all are processed in a manner similar to APP. However, the Aβ sequence is unique in APP, and the amyloid fibrillar form of the Aβ sequence of 40–42 amino acid peptide of which is the primary component of amyloid plaques found in the brains of individuals with Alzheimer's disease and Down's syndrome, can be generated only from APP through its proteolysis. APP and APLP2 are expressed ubiquitously throughout the body with especially abundant expression in the nervous system; whereas APLP1 is reported to be predominantly expressed in the nervous system. Regarding the structure of human APP, the one encoding for the isoform of APP_770_ amino acids that is the longest APP isoform, spans approximately 240 kb and contains 18 exons (for details, see [2],[3]). It has a long N-terminal extracellular domain (EC), and a short C-terminal cytoplasmic region of the intracellular domain (IC), and a short transmembrane domain (TM). The full-length APP is processed by at least three proteinases, termed α, β, and γ-secretases. The amino-acid sequence of the Aβ region is subjected to cleavage by β-secretase (β-site APP cleaving enzyme 1: BACE1) cleaves after Met671 (β), and also by α-secretase cleaves after Lys687 (α). Cleavage by α-secretase or β-secretase yields large soluble APP derivatives (called APPsα and APPsβ, respectively) and generation of α- or β-carboxyl-terminal fragments (APP-CTFα and APP-CTFβ, respectively). The APP-CTFs are subsequently cleaved by γ-secretase in the transmembrane domain, TM, at the position 711: γ40 or 713: γ42, to generate either a 3-kDa product (p3, from APP-CTFα) that is 24 amino acid residues following the cleavage by the γ40 or 26 amino acid residues following the cleavage by the γ42 (non-amyloidogenic pathway) and the APP intracellular domain (AICD) or Aβ peptides (from APP-CTFβ) mainly 40 (cleavage at the position 711 by the γ40), and 42 (cleavage at the position of 713 by the γ42) amino acid residues (amyloidogenic pathway) and AICD. In any way, α-, β-and γ-secretase processing of APP (at the N-and C-terminals of the Aβ sequence) also occur under physiological conditions. This indicates that all fragments of APP, including the Aβ sequence, are part of normal physiology. During transcription, alternative splicing (AS) generates APP-mRNA isoforms that range from 365 to 770 amino acid residues. The major expressed isoforms of APP have 695, 751 or 770 amino acids residues [2],[3]. APP_751_ and APP_770_ isoforms contain a domain homologous to the Kunitz-type serine protease inhibitor (KPI) located in exon 7 of the extracellular sequences, and these isoforms are commonly expressed in non-neuronal cells. APP_695_ isoform lacks the KPI domain and is predominantly expressed in neurons and accounts for the primary source of APP in brain. The raison and functional significance of this apparent tissue-specific alternative splicing is poorly understood.

### 2.2. Putative functions of APP

Because of the central role of the human APP during the development of AD, human APP and its cellular roles as well as its proteolytic processing are in the focus of intensive research. For examples, the cysteine-rich globular domain (E1) (exons 1–5: amino acid residues 18–190) and the helix-rich domain (E2) (exons 9–14: amino acid residues 366–568) regions in the extracellular domain of APP have been shown to interact with extracellular matrix proteins and heparin sulfate proteoglycans, supporting its role in cell-substratum adhesion, cell-cell adhesion, dimerization, ligand-binding and metal-binding. A number of publications have pointed to an important role of the APP extracellular domain in neurite outgrowth and synaptogenesis, both as full-length protein and as a secreted molecule (APPs) following ectodomain-shredding. Furthermore, a role of APP in cell signaling and apoptosis via AICD has been also documented. Thus, APP may exert these activities in both autocrine and paracrine fashions. Concerning the two other human proteins that have a high degree of homology to APP but do not contain the Aβ sequence: APLP1 and APLP2, and in regard to the pathology of AD, although APLP1 and APLP2 do not produce the toxic Aβ peptide, their roles in functioning separately from, but in support of, APP suggest that they may play a role in the development of the disease. Despite the large number of published studies on human APP, the physiologic function and the structure of the entire protein remain largely unclear until now.

### 2.3. Impact on human diseases

Although the amyloid cascade hypothesis has been dominated the field for more than 20 years, and offered a broad framework to explain AD pathogenesis, it is currently lacking in detail, and certain observations do not fit easily with the simplest version of the hypothesis [2],[3]. The most frequently voiced objection is that the number of amyloid deposits in the brain does not correlate well with the degree of cognitive impairment that the patient experienced in life. Furthermore, over recent years, data have illustrated that reciprocal interactions between APP and its various metabolites, including Aβ, can powerfully regulate key neuronal functions including cell excitability, synaptic transmission and neural plasticity [2],[3]. As a consequence, perturbation of some of these activities may contribute to AD pathogenesis. We are entering an era in which the unitary view of AD as a single sequential pathological pathway with Aβ considered as the only initial and causal event is likely to be progressively replaced by a more complex picture in which AD is considered as a multi-parameter pathology that is subtended by several partly independent pathology processes. In this disease, neuronal injury could be caused by different factors, with various possible sequences of pathological events. In contrast to monogenic disease, sporadic AD (SAD) exhibits numerous non-Mendelian anomalies that suggest epigenetic modifications, gene-gene and/or gene-environment interactions in the disease etiology. Compared to genetic causes, epigenetic factors are probably much more suited to explain the observed anomalies in SAD as aberrant epigenetic patterns may be acquired during many developmental stages. The epigenome is particularly susceptible to deregulation during early embryonal and neonatal development, puberty and especially old age, which is the most important known risk factor for AD [2],[3]. Indeed, mutations in familial AD (FAD) represent a very small percentage (∼1%), and ∼99% of cases are SAD [2],[3]. Multiple studies conducted to determine disease-causative loci have revealed that AD is highly complex and heterogeneous in nature. Therefore, non-genetic factors, such as epigenetic modifications, gene-gene and/or gene-environment interactions may also be causative and currently the subject of intense research [2],[3]. Recently, the impact of APP on different human diseases such as neurodevelopmental and neurodegenerative disorders including rare diseases such as autism [5]–[7], FXS [6]–[8], ALS [9], MS [10]–[14], LND [15]–[20]; common and complex disorders such as AD [21]–[31]; metabolic disorders such as diabetes [32]–[34]; and also cancer [35]–[46] has been reported. Overall, there may be many different factors that could cause such diseases such as genetic, epigenetic, epistasis (gene-gene interactions), biologic and environmental factors that act alone or together to influence complex traits, and then the impact of APP (via accumulation of APP in the axons of neurons in MS lesions as well as the correlation of amyloid-β (Aβ) peptide with different stages of MS [10]–[14]; or its cleavage products such as elevated levels of the soluble APP derivative after cleavage by α-secretase: APPsα found in plasma from autistic patients [5]–[7]; APP and APPsα perform a wide array of cellular activities including cell adhesion, migration, neurite outgrowth, and general growth-promoting properties suggesting an important function of APP as a tumor growth factor in the pathogenesis of several somatic tissue cancers [3],[4],[35]–[46]; or via epistasis between mutated hypoxanthine phosphoribosyltransferase 1 (*HPRT1*) and *APP* genes in LND [18]–[20]) on such diseases has been so suggested. Here is an opinion regarding this issue.

## 3. Opinion

Role of epigenetic modifications in gene-gene and/or gene-environment interactions in rare diseases is a key issue in molecular physiology and medicine because the understanding about the mechanisms that explain the influences of epigenetic regulation in rare diseases will provide useful principles for other common and complex disorders. Regarding this issue, the most important discovery was obtained from the research work in LND (a rare X-linked inherited neurogenetic disorder of purine metabolism affecting 1 in 380,000 people, and caused by deficiency of the enzyme hypoxanthine-guanine phosphoribosyltransferase, HGprt, EC. 2.4.2.8; MIM 300800. Complete or severe deficiency of HGprt activity leads to LND that is characterized by an overproduction of uric acid, gout, intellectual impairment, and self-injurious behaviors such as self-biting. Partial deficiency of HGprt enzyme activity is characterized by consequences of overproduction of uric acid and variable spectrum of neurological manifestations, without the self-injurious behaviors: Lesch-Nyhan variants, LNVs, [18]–[20]) in which several APP-mRNA isoforms encoding diver APP protein isoforms ranging from 120 to 770 amino acids (with or without mutations and/or deletions), and APP-mRNA isoforms with a deletion followed by an insertion (INDELS) accounted for epigenetic mechanisms in the regulation of alternative APP pre-mRNA splicing due to epigenetic modifications and/or epistasis as well as for epigenetic control of genomic rearrangements of *APP* gene have been found, for the first time, in fibroblasts from normal subject as well as from LND and LNVs patients [18],[19]. In addition, by using a kinetic method based on RT-PCR technique coupled with direct sequencing for identifying the most abundant APP-mRNA isoform that may be decisive for the normal status or disease risk [20], the defective APP-mRNA isoform of 624 bp, with a deletion starting after 49 bp of the 5′ end of exon 3 followed by a complete deletion of exons 4–15, mutations in exon 1: c.22C > T, p.L8F, and exon 3: c.269A > G, p.Q90R encoding APP_207_ isoform was found from most of LND patients [20]. Then, the results showed that expression of the *APP* gene is under epigenetic regulation caused by genetic and environmental factors as well as life events and aging [3],[18]–[20], and indicated for the first time, a role for epistasis [20],[47]–[49] between mutated *HPRT1* and *APP* genes affecting the regulation of alternative APP pre-mRNA splicing. Hence, APP pathway is possibly implicated in the development of the neurological and behavioral features of LND/LNVs. Epistasis is important, ubiquitous, and has become a hot topic in complex disease genetics in recent years. A gene does not function in isolation and by itself, but rather acts with other genes in a network, to influence complex traits [47]–[49]. It is important to note herein that there were some reported cases suggesting an association between LND and hypercoagulability manifesting as recurrent myocardial infarctions, thromboembolic disease, and thrombus formation [15],[16] while APP is an important regulator of vein thrombosis and controls coagulation and neutrophil extracellular traps (NETs) formation via the KPI-containing soluble APPsα fragment that were demonstrated in vitro to be effective inhibitors of the coagulation FXa, FIXa, FXIa, and FVIIa:tissue factor complex [17]. Theses findings highlight the impact of APP on LND.

Epigenetic changes involve histone and DNA modifications, which can result in drastic phenotypic changes-phenomena that are particularly interesting because these epigenetic events are inherently reversible. Our knowledge about epigenetics is still limited, and some mechanisms have been studied more thoroughly like histone acetylation and DNA methylation, yet much remains to be revealed. Until now, we had identified genetic mutations that could change the epigenetic patterns; but we still do not understand which are the altered putative downstream genes (epigenetically regulated) that result in specific clinical phenotypes. It is important to note herein that epigenetic regulation determines not only what parts of the genome are expressed but also how they are spliced. Alternative splicing (AS) is one of many processes that mediate gene regulation in metazoans. AS is considered to be a key factor underlying increased cellular and functional complexity in higher eukaryotes [50]. Up to 59% of human genes generate multiple mRNA by AS, and ∼80% of AS results in changes in the encoded protein [51], revealing what is likely to be the primary source of human proteomic diversity. During AS of precursor mRNA (pre-mRNA), different combinations of 5′ and 3′ splice site pairs are selected, resulting in the generation of divers mRNA and protein variants. Pre-mRNA splicing takes place within the spliceosome, a large molecular complex composed of five small nuclear ribonucleoproteins (snRNPs) U1, U2, U4, U5, U6, and approximately 50–100 non-snRNP splicing factors [52]. The spliceosome recognizes specific sequences in pre-mRNA to define intron-exon boundaries and to facilitate splicing. The AS can be influenced by both the aging process and/or environmental factors [52]. The chromatin state and epigenetic factors, such as DNA methylation, and histone modifications, can be involved in the splicing process. The structure of the promoter regulating the expression of a gene can affect AS. Variations in promoter sequences can alter gene expression directly by altering a transcription factor-binding site or indirectly by changing the organization of chromatin. Therefore, a misregulation of AS plays a large role in numerous human diseases (for details, see [52]).

The following concepts should be considered: (a) APP is an extremely complex molecule that may be functionally important in its full-length configuration, as well as the source of numerous fragments with varying effects on cellular functions, yet the normal function of APP remains largely unknown. APP as well as all of its fragments including the Aβ sequence, are part of normal physiology. The targeting of the components of APP processing as a pharmacologic strategy will not be without consequences. Therefore, a more complete understanding about its physiological function will not only to provide insights into the pathogenesis of diseases but may also prove vital in the development of an effective therapy; (b) APP, a housekeeping gene and an endogenous ligand (http://www.genenames.org/genefamilies/ENDOLIG) [2],[3],[20], is an important molecular hub at the center of interacting pathways and acts as a permissive factor for various cellular functions, and therefore it is not surprising that altered APP processing may affect neuronal as well as non-neuronal cellular functions through a host of altered cellular and molecular events found in human diseases; (c) To be successful, epigenetic treatments must be selective to irregular cells; otherwise, activating gene transcription in normal cells could make them cancerous, so the treatments could cause the very disorders they are trying to counteract [52]. The identification of molecules capable of correcting and or inhibiting pathological splicing events is therefore an important issue for future therapeutic approaches. To this end, the defective APP-mRNA isoform responsible for the disease in cells and tissues appears as an ideal target for epigenetic therapeutic intervention and antisense drugs are potential treatment [52],[53].
